# *Vibrio cholerae* autoinducer-1 enhances the virulence of enteropathogenic *Escherichia coli*

**DOI:** 10.1038/s41598-019-40859-1

**Published:** 2019-03-11

**Authors:** Orna Gorelik, Niva Levy, Lihi Shaulov, Ksenia Yegodayev, Michael M. Meijler, Neta Sal-Man

**Affiliations:** 1 The Shraga Segal Department of Microbiology, Immunology and Genetics, Faculty of Health Sciences, Ben-Gurion University of the Negev, Beer-Sheva, Israel; 2 The Department of Chemistry and the National Institute for Biotechnology in the Negev, Ben-Gurion University of the Negev, Beer-Sheva, Israel

## Abstract

Diarrhoea is the second leading cause of death in children under the age of five. The bacterial species, *Vibrio cholerae* and enteropathogenic *Escherichia coli* (EPEC), are among the main pathogens that cause diarrhoeal diseases, which are associated with high mortality rates. These two pathogens have a common infection site—the small intestine. While it is known that both pathogens utilize quorum sensing (QS) to determine their population size, it is not yet clear whether potential bacterial competitors can also use this information. In this study, we examined the ability of EPEC to determine *V. cholerae* population sizes and to modulate its own virulence mechanisms accordingly. We found that EPEC virulence is enhanced in response to elevated concentrations of cholera autoinducer-1 (CAI-1), even though neither a CAI-1 synthase nor CAI-1 receptors have been reported in *E. coli*. This CAI-1 sensing and virulence upregulation response may facilitate the ability of EPEC to coordinate successful colonization of a host co-infected with *V. cholerae*. To the best of our knowledge, this is the first observed example of ‘eavesdropping’ between two bacterial pathogens that is based on interspecies sensing of a QS molecule.

## Introduction

Bacteria constantly monitor both their own population sizes and the composition of the different species in their surroundings via a mechanism known as quorum sensing (QS). To facilitate this process, the bacterial cells synthesize and secrete signalling molecules, termed autoinducers (AIs). As the size of a bacterial population increases, the concentration of AIs in that population rises accordingly. Once a threshold concentration has been reached, the AIs bind to specific QS receptors that initiate activation or repression of the transcription of various target genes^1–3^. These genes are involved in a wide range of processes, such as bioluminescence, virulence, biofilm formation, DNA uptake, and sporulation^2–5^, that are beneficial to the bacteria when they take place in a coordinated manner.

The mechanism of QS was initially characterized in the marine species *Vibrio harveyi* and *V. fischeri*^6^ and later in the human species *V. cholerae*^7–9^, the pathogen responsible for the diarrhoeal disease, cholera. It has been shown that at low population densities of *V. cholerae*, virulence and biofilm formation genes are active, while at high *V. cholerae* densities, when AIs concentrations are elevated, these processes are repressed^10–12^. In *V. cholera*, QS is orchestrated through four functionally redundant receptors that detect two AIs, namely, cholera autoinducer 1 (CAI-1) and autoinducer 2 (AI-2)^8,13^. CAI-1 is biosynthesized by the CqsA enzyme and is detected by the CqsS receptor^14,15^. CqsA enzymes exist in all species of *Vibrio*, producing various CAI-1-like moieties that have different acyl chain lengths and contain various modifications. The CqsS receptors in the different species of *Vibrio* respond to many CAI-1-like molecules with different affinities. It has thus been assumed that CAI-1 functions as an intra-genus communication signal^3,7^. In contrast, AI-2 is produced and detected by a wide variety of bacteria, such as *Salmonella* species and *Escherichia coli*, and is therefore believed to be involved in inter-species signalling^16^.

Although CAI-1 and AI-2 have different cellular receptors, their activity is coordinated, as all the receptors inactivate the LuxO protein upon ligand binding^13,17^. Active LuxO represses the expression of HapR, which is a negative regulator of virulence and biofilm formation genes and a positive regulator of hap protease expression^9^. Consequently, when the density of a bacterial population is low, the AIs are diluted in the extracellular environment and LuxO is active. HapR levels are accordingly low, and the expression of virulence and biofilm formation genes is high^8,11,13,18^. At high bacterial population densities, the concentration of the AIs increases, resulting in activation of the QS receptors and repression of LuxO. The HapR expression level is raised, resulting in the repression of virulence and biofilm formation genes^8,11,13,18^. This multi-factor coordination is critical to the ability of *V. cholerae* to colonize its host when the bacterial population density is low and to adapt bacteria for transmission once it becomes critically high.

A pathogen that shares a common infection site with *V. cholera* is the bacterial species enteropathogenic *E. coli* (EPEC). This bacterial pathogen, similarly to *V. cholerae*, colonizes the small intestine and causes severe diarrhoea, mostly in infants^19^. EPEC utilizes several QS systems to monitor cell density. One such system is a variant of the LuxI/R system, which is used by most Gram-negative bacteria. This system was first discovered in *V. fischeri*^20^, although, notably, it is not present in *V. cholerae*. LuxI is responsible for the synthesis of N-acyl-homoserine-lactones (AHLs), while LuxR is the AHL receptor. *E. coli* strains, including EPEC, lack the *luxI* gene and therefore cannot synthesize AHLs^21^. However, they encode the SdiA protein, a LuxR homolog that recognizes a wide range of AHLs synthesized by other bacterial species and respond to it by activating acid resistance genes^22^ and repressing virulence genes^23^.

An additional QS system found in EPEC is the AI-2 system. While some studies have reported that the AI-2 system acts on virulence and motility genes^24–27^, more recent studies have suggested that virulence and motility are controlled by a different signalling molecule, designated AI-3^28,29^. Indeed, the AI-3/epinephrine/norepinephrine QS system has been described in a pathogen related to EPEC, namely, enterohaemorrhagic *E. coli* (EHEC). It has been shown in EHEC that this QS system is involved in the communication between prokaryotic and eukaryotic cells^28,29^ and that AI-3 and epinephrine activate the transcription of virulence and flagellar genes^28^. We note here that the influence of AI-2 on the virulence mechanisms of *E. coli* pathogens is thus still unclear.

The best studied QS-regulated virulence mechanism is the type III secretion system (T3SS). The T3SS is used by many Gram-negative pathogens to deliver effector proteins from the bacterial cell directly into the host cell cytoplasm. These effector proteins manipulate key intracellular pathways, which ultimately promote bacterial replication and transmission^30–32^. The T3SS structural proteins are encoded in a 35-kbp chromosomal pathogenicity island, termed the locus of enterocyte effacement (LEE)^33^. The LEE comprises 41 genes that are mainly organized in five operons. LEE1, LEE2, and LEE3 contain genes that encode mainly T3SS structural proteins; LEE4 genes encode secreted proteins; and LEE5 genes encode proteins responsible for the intimate association of the bacteria with the host cell^34^.

In this study, we examined whether, in the presence of *V. cholerae*, EPEC modulates its virulence in response to the population size of *V. cholerae*. We indeed observed an enhancement of EPEC virulence when EPEC was grown in co-culture with *V. cholerae* or in the presence of a filtered supernatant from a *V. cholerae* culture. Using a synthetic CAI-1, the primary *V. cholerae* AI, we showed that EPEC responded to CAI-1, even though it cannot itself produce this AI. Surprisingly, we found that EPEC virulence genes were upregulated in response to elevated CAI-1 concentrations, in contrast to *V. cholerae*, in which high concentrations of CAI-1 are known to lead to the repression of virulence genes^8,11,13,18^. It is likely that this sensing and virulence upregulation response of EPEC allows it to determine the virulence status of *V. cholerae* and hence to upregulate its virulence when *V. cholerae* virulence is downregulated, thereby optimizing the timing for successful colonization of the small intestine.

## Results

### EPEC alters its T3SS activity when grown in co-culture with *V. cholerae*

To reveal the cross-talk between *V. cholerae* and EPEC, both of which colonize the small intestine, we cultured wild-type (WT) EPEC in the presence of *V. cholerae* and then examined the EPEC T3SS activity. To support both *V. cholerae* growth and the induction of EPEC T3SS, which requires growth conditions that simulate those in the human gastrointestinal tract^35^, the bacterial strains were grown statically in a 1:1 (v/v) mixture of Dulbecco’s modified Eagle’s medium (DMEM) and Luria-Bertani (LB) medium in a tissue culture incubator (with 5% CO_2_). We evaluated EPEC T3SS activity by determining the levels of two EPEC translocators, EspA and EspB, found in the bacterial supernatants. We also determined the expression levels of EscJ, which is a structural component of the T3SS apparatus, and of the effector protein Tir, which is expected to be retained within the bacterial cells in the absence of host cells. Plain LB, without a bacterial inoculum, and co-culture of EPEC with *E. coli* DH10B strain were used as controls.

**Figure 1:**
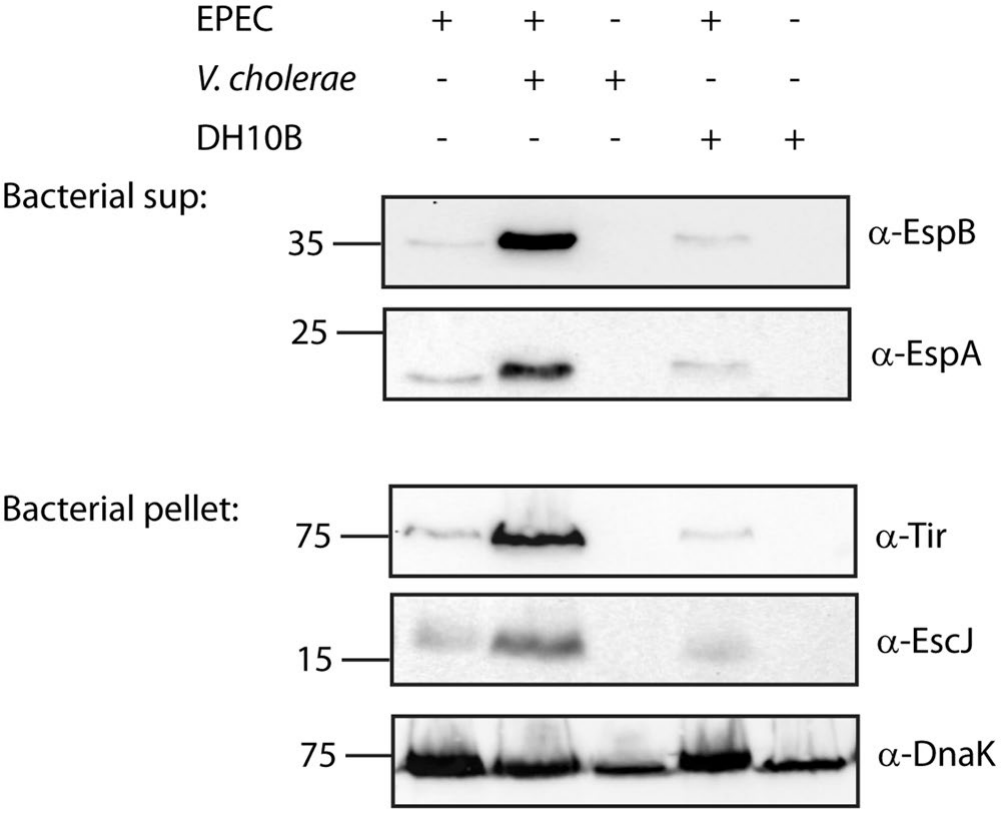
A mixed culture of EPEC and *V. cholerae* shows elevated T3SS activity compared to an EPEC pure culture. Pure overnight cultures of EPEC, *V. cholerae*, and *E. coli* DH10B were sub-cultured into fresh 1:1 (v/v) DMEM:plain LB mixture as pure or mixed cultures. The cultures were grown under semi T3SS-inducing conditions for 6 h, and then bacterial pellets and supernatant (sup) were separated and analyzed. The secreted proteins were concentrated from supernatants of bacterial cultures and analyzed by 12% SDS-PAGE and western blot analysis using anti-EspB and anti-EspA antibodies for the secretion of the EspA and EspB translocator proteins. The expression of the structural T3SS protein, EscJ, and the effector protein, Tir, which should remain mostly within the bacterial cytoplasm at this stage, were analyzed by subjecting the bacterial pellets to SDS-PAGE and western blot analysis using an anti-EscJ and anti-Tir antibodies. Samples were also probed with anti-DnaK to demonstrate equal loading.

The supernatant fraction of the EPEC and *V. cholerae* co-culture showed elevated levels of EspA and EspB compared to their levels in the supernatant of an EPEC pure culture (Fig. 1). As expected, the supernatant of the *V. cholerae* pure culture was negative for both EspA and EspB, thereby excluding the possibility that the anti-EspA and anti-EspB antibodies react with a *V. cholerae* component. Examination of the expression levels of EscJ and Tir within the bacterial pellets showed higher expression of the two T3SS proteins in the co-culture sample of EPEC and *V. cholerae* relative to the pure culture of EPEC. The bacterial pellet of the *V. cholerae* pure culture was negative for anti-EscJ and anti-Tir, thereby confirming that these antibodies are specific to EPEC T3SS components. DnaK levels within the bacterial pellets demonstrated equal loading (Fig. 1). To determine whether the T3SS-related virulence of EPEC is specifically upregulated in response to *V. cholerae*, we co-cultured WT EPEC with *E. coli* DH10B and examined EPEC T3SS activity. The supernatant of the EPEC and *E. coli* DH10B co-culture showed levels of T3SS translocators similar to those in the supernatant of the EPEC pure culture, while no signal was observed for the *E. coli* DH10B pure culture (Fig. 1). In addition, the bacterial pellet of the EPEC and *E. coli* DH10B co-culture showed expression levels of EscJ and Tir similar to those of the EPEC pure culture. These findings suggest that the T3SS activity of EPEC is upregulated specifically in response to *V. cholerae* and not simply in response to the presence of another bacterial strain.

Finally, to show that the upregulated T3SS activity observed in the co-culture of EPEC and *V. cholerae* was not due to a higher bacterial count of EPEC, we determined the number of colony forming units (CFUs) in both co-cultures and pure cultures. To facilitate separation between EPEC and *V. cholerae* or *E. coli* DH10B, we grew the bacteria in Transwells (Merck MultiScreen filter plate 0.22 μm), which allow the passage of molecules but not bacterial cells. After 6 h growth in 1:1 (v/v) DMEM:plain LB mixture, EPEC was collected and spotted at 10-fold serial dilutions on LB plates containing streptomycin. We observed a ~100-fold decrease in EPEC CFUs when EPEC was co-cultured with either *V. cholerae* or *E. coli* DH10B relative to the number of CFUs in an EPEC pure culture (Fig. S1). These results confirmed that the upregulation of the T3SS activity observed in the EPEC and *V. cholerae* co-culture sample was not due to higher EPEC counts.

### Altered T3SS activity of EPEC in response to exposure to a *V. cholerae* supernatant

**Figure 2:**
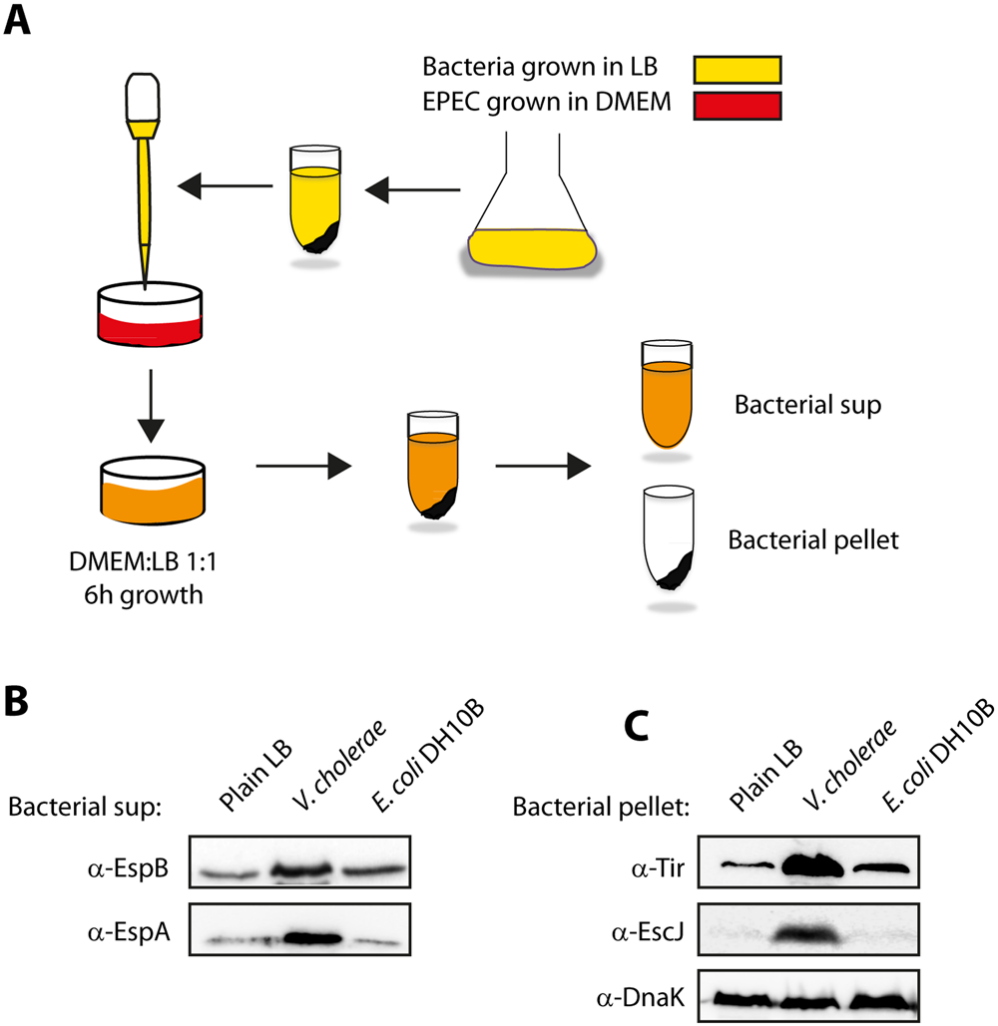
EPEC grown in the presence of a *V. cholerae* supernatant shows elevated T3SS activity. (**A**) Scheme of the experiment: WT EPEC was grown in DMEM medium (red) in the presence of supernatants of bacteria grown in LB or plain LB (yellow). After 6 h of growth, the bacterial EPEC pellets and supernatants (sup) were separated and analyzed for T3SS activity. (**B**) WT EPEC strain was grown in DMEM medium mixed 1:1 (v/v) with plain LB, *V. cholerae* supernatant or *E. coli* DH10B supernatant for 6 h. The secreted proteins were then concentrated from supernatants of bacterial cultures and analyzed by 12% SDS-PAGE and western blot analysis using anti-EspB or anti-EspA antibodies (translocators). While low secretion of EspB and EspA were detected for EPEC grown in DMEM medium mixed 1:1 (v/v) with plain LB or in the presence of *E. coli* DH10B supernatant, high secretion of EspB and EspA were observed for WT EPEC grown in the presence of *V. cholerae* supernatant. (**C**) Whole cell lysates were analyzed for the expression of the structural T3SS protein, EscJ, and the effector protein Tir by loading the bacterial samples on SDS-PAGE and performing western blot analysis using anti-EscJ and anti-Tir antibodies. While low expression of EscJ and Tir were detected for EPEC grown in DMEM medium mixed 1:1 (v/v) with plain LB or in the presence of *E. coli* DH10B supernatant, high expression of EscJ and Tir was observed for WT EPEC grown in the presence of *V. cholerae* supernatant. Samples were also probed with anti-DnaK antibody to confirm equal loading of lysates.

To determine whether the T3SS activity of EPEC is upregulated in response to *V. cholerae* signalling molecules secreted into the *V. cholerae* growth medium, we cultured *V. cholerae* in LB medium overnight and separated the supernatant from the bacterial cells by centrifugation and filtration. To assess whether EPEC can detect the signalling molecules of *V. cholerae* and respond to them by alteration of its T3SS activity, we added a purified *V. cholerae* supernatant to the growth medium of WT EPEC in a 1:1 ratio (Fig. 2A). Plain LB and the supernatant of an *E. coli* DH10B culture were used as controls.

WT EPEC grown in a 1:1 ratio of DMEM:*V. cholerae* culture supernatant showed elevated levels of translocator secretion (EspA and EspB) relative to EPEC grown in a 1:1 (v/v) DMEM:plain LB mixture or in a DMEM:*E. coli* DH10B culture supernatant (Fig. 2B). A similar upregulation pattern was observed for the expression of the T3SS proteins, EscJ and Tir, when WT EPEC was grown in DMEM:*V. cholerae* culture supernatant relative to growth in DMEM:plain LB or DMEM:*E. coli* DH10B culture supernatant, all in a 1:1 ratio (Fig. 2C). DnaK levels demonstrated equal loading of bacterial lysates. These findings suggested that the T3SS activity of EPEC is upregulated in response to secreted components present in the supernatant of *V. cholerae* cultures.

### Effect of synthetic CAI-1 on EPEC virulence and growth

**Figure 3:**
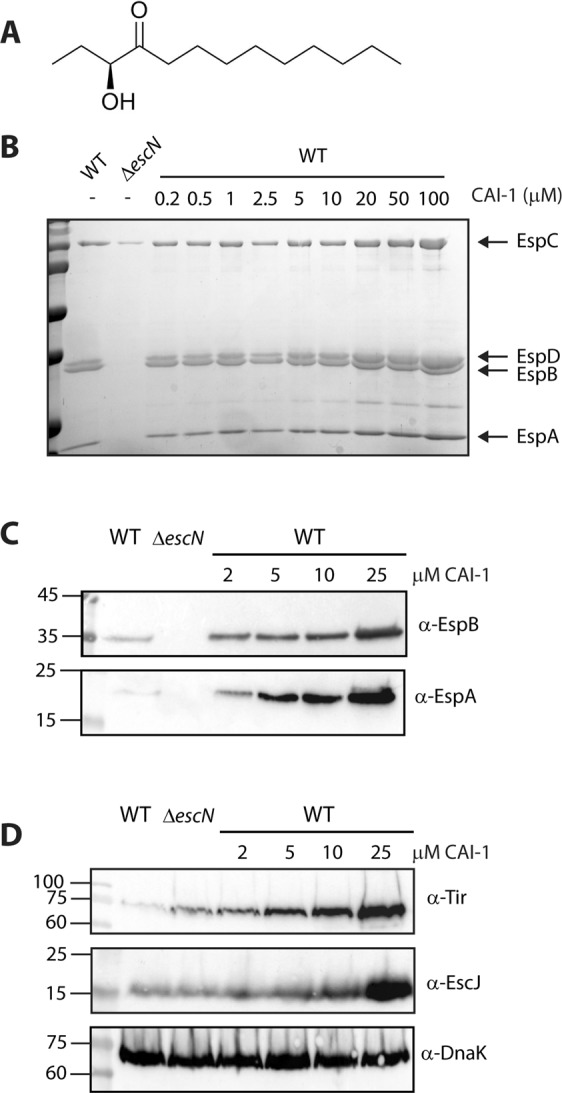
CAI-1 enhances T3SS activity of EPEC in a dose-dependent manner. (**A**) Chemical structure of CAI-1. (**B**) Synthetic CAI-1 was added (at concentrations of 0.2–100 μM) to WT EPEC grown under T3SS-inducing conditions (DMEM) for 6 h. The secreted proteins were concentrated from the supernatants of the bacterial cultures and analyzed by 12% SDS-PAGE and Coomassie blue staining. The Δ*escN* mutant strain, which is unable to secrete through the T3SS, was used as a negative control. The locations of the type III translocators EspA, EspB, and EspD are indicated to the right of the gel. Also indicated is the location of EspC, which an autotransporter that is also secreted during EPEC infection. We observed that addition of CAI-1 at a concentration of 5 μM or higher enhanced EPEC T3SS activity. (**C**) and (**D**) WT EPEC was grown in 1:1 (v/v) DMEM:plain LB mixture for 6 h in the presence or the absence of synthetic CAI-1 (2, 5, 10 and 25 μM). The secreted proteins were concentrated as described above and analyzed by SDS-PAGE and western blot analysis using anti-EspB and anti-EspA antibodies (**C**) while the bacterial pellets were analyzed by SDS-PAGE and western blot analysis using anti-Tir, anti-EscJ and anti-DnaK antibodies (**D**).

Since CAI-1 is the primary QS molecule of *V. cholerae*^7^, we examined whether the addition of synthetic CAI-1 (for chemical structure, see Fig. 3A) would induce an effect on the T3SS activity of EPEC similar to that of the *V. cholerae* supernatant or to co-culture with *V. cholerae* (the relative levels of CAI-1 in the supernatants of *V. cholerae* and *E. coli* DH10B are presented in Fig. S2). To this end, we synthesized CAI-1 and examined its effect on the T3SS activity of WT EPEC. In this set of experiments, we grew WT EPEC strain in DMEM alone, as is commonly done when examining the T3SS activity of EPEC. We added to these WT cultures micromolar concentrations of CAI-1, based on the concentrations of CAI-1 under natural conditions, which vary between ≤1 μM^7^ in planktonic cultures of *V. cholerae* and 32 μM in *V. cholerae* biofilms^36^. The secreted proteins were analyzed by loading the concentrated supernatants onto SDS-PAGE and visualizing all the T3SS translocators (EspA, EspB, and EspD) by Coomassie staining. The Δ*escN* EPEC strain, a T3SS ATPase mutant that is devoid of T3SS activity, was used a negative control. When CAI-1 was added to the WT EPEC culture at concentrations of 5 μM or higher, we observed elevated secretion of T3SS components into the extracellular medium compared to a sample supplemented with DMSO alone (the solvent for the stock solution of CAI-1, 1% (v/v); Fig. 3B). This result suggests that the elevated EPEC T3SS activity that we observed for EPEC and *V. cholerae* co-culture and for the EPEC strain grown in the presence of *V. cholerae* supernatant are most probably related to the ability of EPEC to detect and respond to CAI-1—whether natural or synthetic. To better observe the effect of synthetic CAI-1 on EPEC, we added 2, 5, 10 and 25 μM CAI-1 to WT EPEC grown in 1:1 DMEM:plain LB. These conditions do not induce a maximal T3SS response and therefore allow better detection of T3SS upregulation activity. Analysis of the supernatants of WT EPEC in the presence of synthetic CAI-1 showed dose-dependent elevation of EspA and EspB relative to WT EPEC supplemented only with 0.25% (v/v) DMSO (Fig. 3C). A similar dose-dependent pattern was observed for the expression of Tir and EscJ within the bacterial pellets of WT EPEC grown in the presence of CAI-1 (Fig. 3D). We estimate that the CAI-1 concentration in the *V. cholerae* supernatant (Fig. 2B,C) was 10–25 μM, based on the EspA, EspB, Tir and EscJ levels observed when WT EPEC was incubated with known concentrations of CAI-1 (Fig. 3C,D).

**Figure 4:**
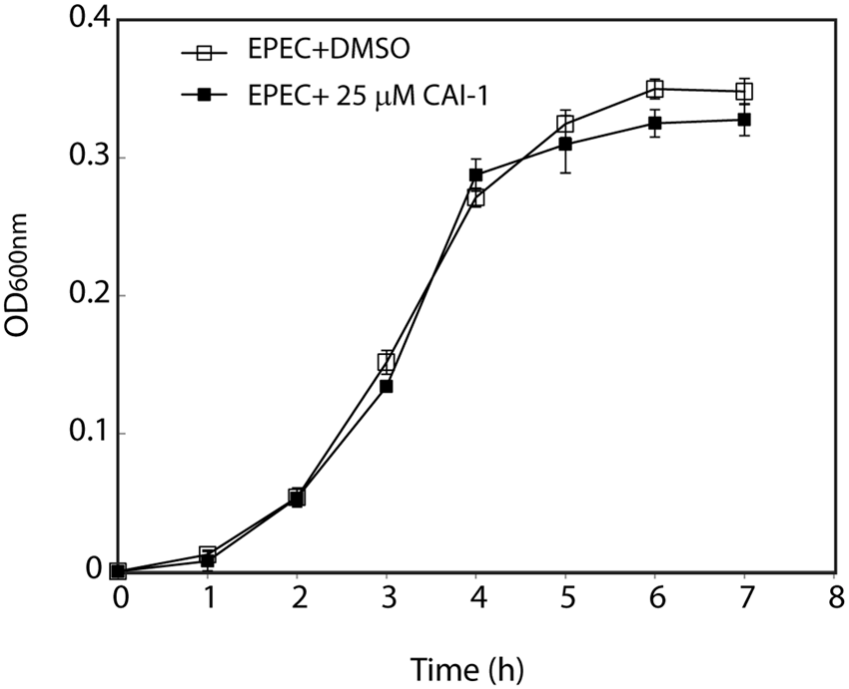
CAI-1 does not affect EPEC growth. WT EPEC growth was monitored in the presence of 25 μM CAI-1 or a DMSO supplement by measuring the optical density at 600 nm of the bacterial samples. The bacteria were grown statically in a 1:1 DMEM:plain LB mixed medium in a CO_2_ incubator. Growth curves were determined in triplicate.

To examine whether the effect of CAI-1 on EPEC T3SS activity is related to the effect of the AI on bacterial growth, we monitored the optical density of WT EPEC samples grown under T3SS inducing conditions [static growth in 1:1 (v/v) DMEM:plain LB; 5% CO_2_; Fig. 4) and non-T3SS inducing conditions (LB medium; shaking; data not shown) in the presence of 25 μM CAI-1 or 0.25% (v/v) DMSO. Regardless of the growth conditions, we did not observe any effect of CAI-1 on the growth of EPEC (Fig. 4 and data not shown). This result contradicts the findings of other studies showing that bacterial AIs, such as CAI-1, play a role in slowing down the growth rate as the bacterial culture enters the stationary phase^27,36,37^. Here, we observed a specific case in which the bacteria did indeed sense the presence of the AI in the medium, but reacted to it with an induction of virulence mechanisms but not with a retardation in growth rate. Examination of the effect of CAI-1 on EPEC motility and biofilm formation, which were previously shown to be affected by QS^24^, revealed no effect of CAI-1 on these factors (Fig. S3).

### Effect of synthetic CAI-1 on the transcription of T3SS genes

**Figure 5:**
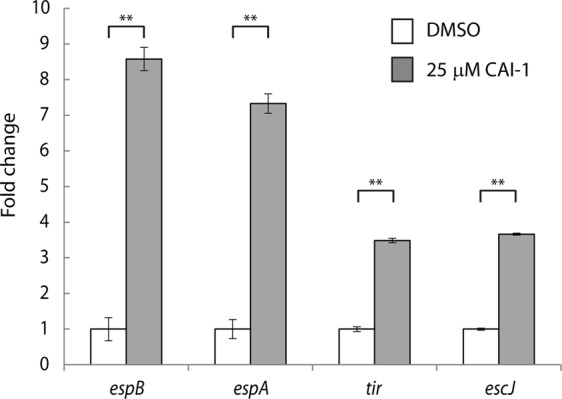
CAI-1 promotes the expression of EPEC virulence genes. WT EPEC was grown statically in 1:1 DMEM:plain LB medium in a CO_2_ incubator in the presence (25 μM) or the absence [0.25% (v/v) DMSO supplement alone] of CAI-1 for 2 h. mRNA levels of *tir*, which encodes the main effector protein secreted by the T3SS, of *espA* and *espB*, which encode T3SS translocator proteins, and of *escJ*, which encodes a structural T3SS protein, were measured by qRT-PCR. mRNA levels of WT EPEC grown in the presence of CAI-1 (gray bars) are presented relative to those of WT EPEC grown in the absence of CAI-1 (white bars). Data are the means of at least three experiments; bars represent standard error; **P < 0.005.

Since CAI-1 is known to act through gene regulation in *V. cholerae*^11,17,18,38,39^, we examined whether addition of CAI-1 to a WT EPEC culture would enhance EPEC virulence through upregulation of T3SS genes. To this end, we cultured WT EPEC under semi-optimal T3SS-inducing conditions [static growth in 1:1 (v/v) DMEM:plain LB, 5% CO_2_] in the presence (25 μM) or the absence [0.25% (v/v) DMSO supplement] of CAI-1 and evaluated the transcription levels of four representative T3SS genes that are encoded on different operons within the LEE, namely, *tir*, which encodes the first translocated effector of the T3SS (LEE5), *espA* and *espB*, the genes that encode the T3SS translocators (LEE4), and *escJ*, which encodes a T3SS structural protein (LEE3). We detected significantly elevated levels of all four genes in the presence of CAI-1 (Fig. 5). These results suggest that CAI-1 enhanced EPEC virulence by upregulating T3SS gene expression. Since no CqsS orthologue has been reported to date in *E. coli* strains, the mechanism of CAI-1 signal transduction in *E. coli* remains unresolved.

### Effect of synthetic CAI-1 on the ability of EPEC to translocate proteins into host cells

**Figure 6:**
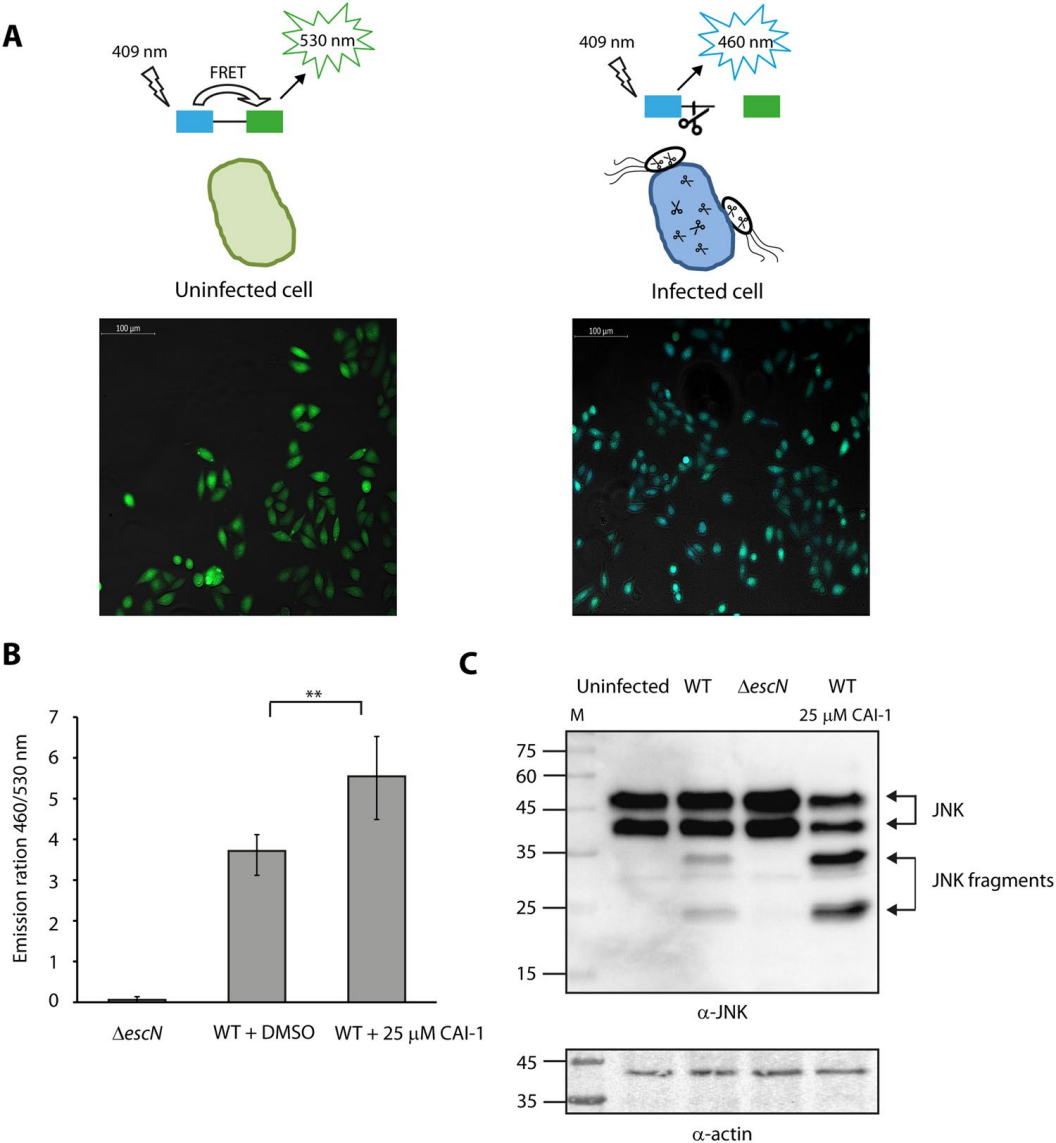
CAI-1 increases EPEC translocation activity into HeLa cells. (**A**) Scheme of the effector translocation assay. HeLa cells loaded with CCF2‐AM emit green fluorescence (530 nm) when excited at wavelength of 409 nm. However, when the fusion protein of EspZ with β‐lactamase (EspZ-TEM) translocates into HeLa cells via the T3SS of WT EPEC, it mediates cleavage of CCF2‐AM, which induces a shift from green to blue fluorescence (460 nm). Representative images of HeLa cells either uninfected or infected with WT EPEC and loaded with CCF2-AM were imaged by fluorescence microscopy and presented as merged blue/green emission images. (**B**) WT EPEC carrying pEspZ-TEM was grown in the presence of 0.25% (v/v) DMSO or 25 μM CAI-1. Effector translocation levels are defined as the ratio between blue (460 nm) and green (530 nm) fluorescence. Data are the means of at least three experiments; bars represent standard error; **P < 0.005. (**C**) HeLa cells were infected with WT EPEC in the presence or absence of 25 μM CAI-1. After 1 h, cells were washed, and host cell proteins were extracted and subjected to western blot analysis using anti-JNK and anti-actin (loading control) antibodies. JNK and its degradation fragments are indicated at the right of the gel. WT EPEC showed degradation of JNK compared to the uninfected sample and the samples infected with Δ*escN*. HeLa cells infected with WT EPEC in the presence of 25 μM CAI-1 showed an enhanced JNK degradation profile compared to the WT EPEC without CAI-1, thus suggesting that CAI-1 promotes the ability of EPEC to translocate effectors into host cells.

To evaluate the ability of CAI-1 to enhance EPEC virulence, we examined the level of the effectors’ translocation from WT EPEC into host cells in the presence or absence of CAI-1. For this purpose, we used the translocation assay developed by Charpentier and Oswald^40^. In brief, EPEC strains carrying an EspZ-TEM-1 chimeric protein were grown under T3SS-inducing conditions with or without CAI-1 and then used to infect HeLa cells for 90 min. Thereafter, the HeLa cells were washed and stained with CCF2/AM, which is a TEM-1 β-lactamase substrate. When excited at wavelength of 409 nm, uninfected HeLa cells exhibited green fluorescence, indicating the absence of β-lactamase activity, while HeLa cells infected with the WT EPEC exhibited blue fluorescence, with the green to blue shift (530 → 460 nm) being caused by CCF2 cleavage by the EspZ-TEM-1 chimeric protein translocated into the host cells by the T3SS of WT EPEC (Fig. 6A). Similarly, we observed that WT EPEC grown in the presence of 25 μM CAI-1 exhibited a significantly elevated level of effector translocation compared to the same strain grown without CAI-1 [with 0.25% (v/v) DMSO supplement alone] (Fig. 6B). As expected, the *escN* null mutant, which is a T3SS ATPase mutant, exhibited very low translocation ability, due to its inability to secrete and translocate effector proteins (Fig. 6B). These results suggest that high levels of CAI-1 are detected by WT EPEC and prime the bacteria for virulence.

To confirm the effect we observed for CAI-1 on the translocation activity of EPEC, we performed an additional set of experiments using an alternative assay. For these experiments, we modified the method developed by Baruch *et al*., which monitors the activity of the natural EPEC effector known as NleD upon its translocation into the host cells, where it cleaves the host protein JNK^41^. To determine the effect of CAI-1 on the transformation activity of EPEC, we infected HeLa cells with WT EPEC in the presence of 25 μM CAI-1 or 0.25% (v/v) DMSO and examined the cleavage pattern of JNK. To allow detection of enhanced translocation activity, we used a short infection time, which prevented full degradation of cellular JNK by WT EPEC. The HeLa culture infected with WT EPEC showed a clear degradation of JNK, in contrast to the uninfected HeLa sample and the sample infected with the Δ*escN* mutant strain (Fig. 6C). However, when we infected HeLa cells with WT EPEC that had been incubated with 25 μM CAI-1, we observed higher level of JNK degradation compared to infection of WT EPEC without CAI-1 (Fig. 6C), thereby providing further support for the notion that CAI-1 enhances EPEC virulence.

### Effect of synthetic CAI-1 on EHEC and *Salmonella* type III secretion

**Figure 7:**
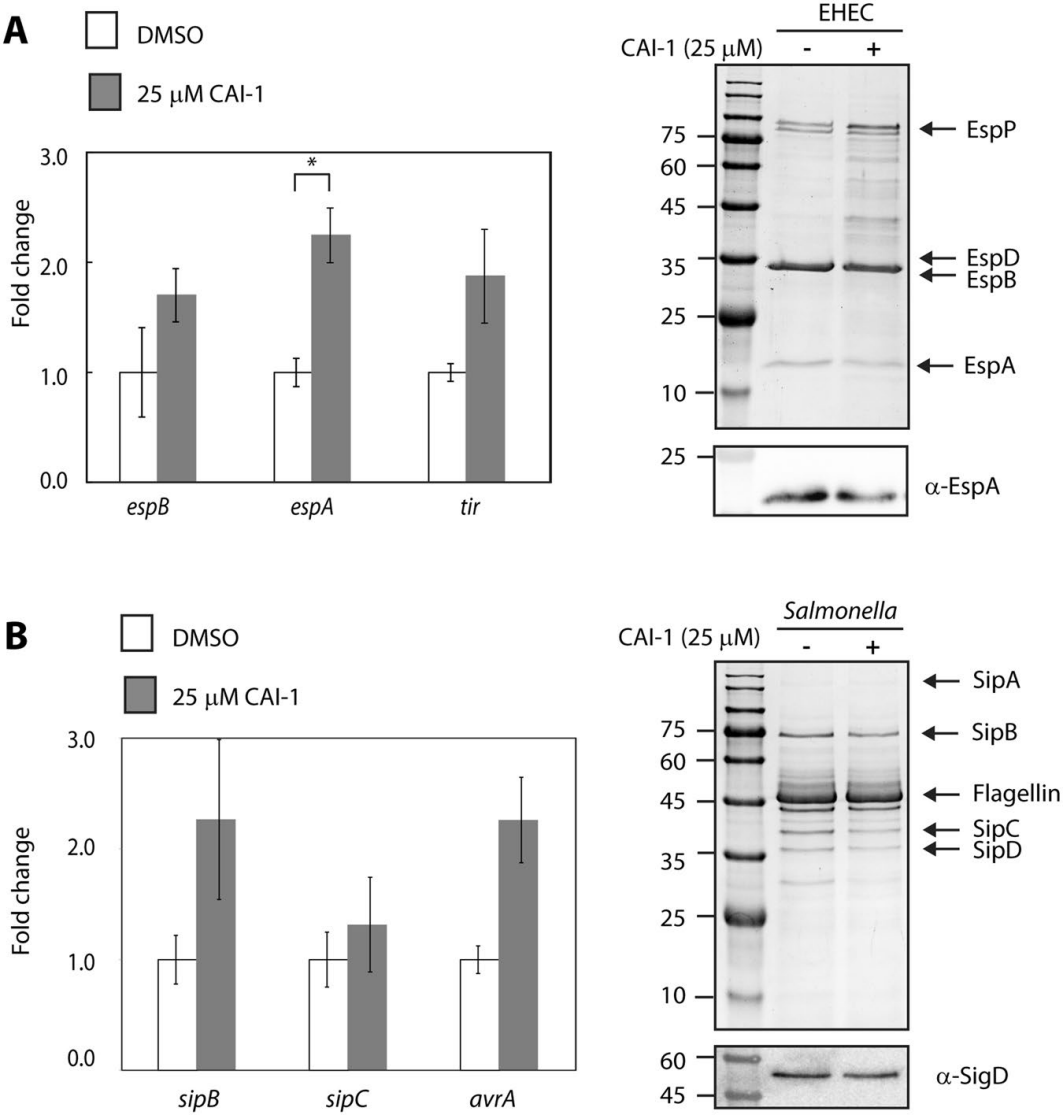
Effect of CAI-1 on the T3SS activity of EHEC and *Salmonella*. Protein profiles of concentrated supernatants of EHEC O157:H7 (**A**) and *S. enterica* serovar Typhimurium SL1344 (**B**), grown under T3SS-inducing conditions in the presence (25 μM) and absence of CAI-1 for 6 h. The secreted proteins were analyzed by 12% SDS-PAGE and Coomassie blue staining or western blot analysis using anti-EspA (EHEC) or anti-SigD (*Salmonella*). The arrows at the right side of the gels indicate known proteins secreted under T3SS-inducing conditions. In addition, mRNA levels of *espA, espB* and *tir* in EHEC (**A**) and of *sipB*, *sipC*, and *avrA* of *Salmonella* (**B**) were measured by qRT-PCR. The mRNA levels of bacteria grown in the presence of CAI-1 (gray bars) are presented relative to those for the bacteria grown in the absence of CAI-1 (white bars). Data are presented for a representative experiment (out of at least three experiments); bars represent standard error; *P > 0.01.

To determine whether the CAI-1 detection and response mechanism is found in other gastrointestinal bacterial pathogens, we examined the effect of CAI-1 on the T3SS activity of EHEC O157:H7 and *Salmonella enterica* serovar Typhimurium (SL1344). For that purpose, we cultured the bacterial strains under T3SS-inducing conditions^42,43^ in the presence of CAI-1 (25 μM) or 0.25% (v/v) DMSO, and collected and analyzed their supernatants by Coomassie blue staining or by western blot analysis using an anti-EspA antibody for EHEC or anti-SigD for *Salmonella*. No obvious effect was observed on the EHEC or *Salmonella* T3SS secretion levels (Fig. 7). To examine whether the addition of CAI-1 to EHEC or *Salmonella* cultures upregulated their T3SS genes, we extracted RNA from the bacteria grown in the presence or absence of CAI-1 and analyzed the transcription levels of three representative T3SS genes (*espA*, e*spB* and *tir* in EHEC and *sipB*, *sipC*, and *avrA* in *Salmonella*). A twofold increase of transcription was detected only for the *espA* gene of EHEC in the presence of CAI-1 (Fig. 7A). These results suggest that the ability to sense and respond to the *V. cholerae* QS molecule, CAI-1, is not ubiquitously distributed among all human intestinal bacterial pathogens.

## Discussion

CAI-1 is the primary AI of *V. cholerae*, and together with AI-2, it regulates the transcription levels of virulence and biofilm formation genes. This process ensures the coordinated behavior of *V. cholera*e populations that optimizes the ability of the pathogen to infect the small intestine or move on to another host when conditions are optimal. However, the small intestine does not play host to *V. cholerae* alone: in addition to the commensal microbiota, other pathogens, such as EPEC, may also be present in the small intestine. Therefore, in order to successfully colonize their host, intestinal pathogens need to monitor both the environmental conditions, which promote bacterial adaptations to the specific niches, and the presence of other – competing or cooperating – pathogens, which will affect their chances to successfully colonize the host.

In this study, we examined whether EPEC can sense and respond to the presence of *V. cholerae*. We hypothesized that if such a sensing and responding mechanism does exist, it would promote upregulation of virulence genes in the presence of the competing bacterial species so as to enable successful competition. Surprisingly, we found that EPEC virulence is upregulated when the concentration of CAI-1 indicates that *V. cholerae*’s virulence is actually downregulated. This mechanism probably allows optimization of the ability of EPEC to colonize its host just as *V. cholerae* prepares to leave it. Recently – most probably due to improved technologies for bacterial diagnosis in stool samples – it has indeed become evident that in patients with diarrhoeal disease there is high prevalence of mixed infections with two or more agents^44–46^. Similarly, it has been reported that 15–30% of patients hospitalized with diarrhoeal diseases are concomitantly infected with *V. cholerae* and EPEC or enterotoxigenic *E. coli*, a related *E. coli* strain that also infects the small intestine^47,48^.

We observed a mild increase of EPEC virulence at CAI-1 concentrations representative of those in planktonic cultures (~2.5–5 μM) and a more pronounced effect when concentrations were similar to the concentration measured in *V. cholerae* biofilms (~30 μM)^7,36^. Although microscopic examination of *V. cholerae* in the intestinal mucosa of rabbits and two-photon microscopy studies of mouse intestines revealed *in-vivo* formation of biofilm aggregates of *V. cholerae*^49,50^, the exact CAI-1 concentration within the small intestine biofilm is unknown.

A recent study that examined the cross-talk between human gut microbiota and *V. cholerae* found that *Ruminococcus obeum*, which is a member of the healthy individual’s microbiota, represses *V. cholerae* colonization by causing *V. cholerae* virulence genes to be downregulated^51^. That study suggested that *R. obeum* synthesizes AI-2 molecules that repress virulence mechanisms and colonization factors of *V. cholerae*. Although this result was to be expected, since AI-2 is synthesized and detected by *V. cholerae* through its LuxP/Q system, the researchers suggested that AI-2 acts through an as-yet uncharacterized mechanism, since the colonization levels of Δ*luxP* and WT *V. cholerae* were not significantly different in the presence and absence of *R. obeum*^51^. Other studies have reported that AI-2 produced by host microbiota species upregulates several major virulence genes of *Pseudomonas aeruginosa*, which does not produce AI-2 itself^52,53^. Those studies therefore demonstrated that the QS communication between host microbiota and bacterial pathogens is inhibitory in nature, whereas the communication between bacterial pathogens revealed by our results was designed to promote bacterial infection.

While it has been suggested that AI-2 functions as an interspecies communication signal, CAI-1 is commonly considered to function mainly as an intra-genus communication signal, since the CqsA/CqsS system is almost exclusively restricted to *Vibrio* species and is highly conserved in this genus^7,15^. CqsA/CqsS displays low signal discrimination, and multiple CAI-1 variants can activate these circuits at different intensities. Here, we found that EPEC can detect and respond to both endogenic and synthetic CAI-1. Nevertheless, since homologues of the CAI-1 synthase (CqsA) or the CAI-1 receptor (CqsS) have never been reported in EPEC, further investigation is required to identify the proteins that are involved in the response to CAI-1 in EPEC. It is possible that EPEC does have a receptor to CAI-1, but not the synthase enzyme, similarly to the LuxR/I system in *E. coli*. Such a receptor would allow EPEC to respond to CAI-1 without being able to produce it.

QS in different strains of *E. coli*, which has mostly been studied in EHEC, evokes a wide range of responses to AIs. These include upregulation of virulence genes by the self-produced AI-3 and the host hormones epinephrine or norepinephrine^26–28,54^, while the *E. coli* AI-1 has the reverse effect on virulence and represses gene expression^23^. This wide range of responses allows the bacterial cells to respond to complex environments and integrate large amounts of information regarding the density of the *E. coli* bacterial population and the composition of bacterial and host cells. In this study, we found that this complexity is not merely a function of the composition of QS molecules in the extracellular medium, as the same molecule, CAI-1, can induce contradictory responses in different bacterial species (e.g., *V. cholerae* versus EPEC). This observation suggests that bacterial communication is dependent not only on the nature of the AIs but also on the particular responding bacteria.

Our findings that CAI-1 upregulates virulence in EPEC but not in the closely related *E. coli* strain, EHEC, or the gastroenteritis pathogen, *Salmonella*, suggest that the ability to detect and respond to CAI-1 has evolved in EPEC due to its common niche – the small intestine – with *V. cholerae*. The ability of EPEC to detect both the presence and the virulence status of *V. cholerae* has possibly improved the fitness of EPEC, as this ability reduces its competition with *V. cholerae* and enables it to time its infection so as to increase its chances to successfully colonize its host. This strategy of a QS-based-mechanism that allows coordination between potentially competing bacterial pathogens may well be a broader phenomenon that occurs in other pathogens that infect common environmental niches, and presents enticing new research avenues.

## Materials and Methods

### The bacterial species, deletion mutant and plasmid

The bacterial species and the plasmid used in this study are listed in Table 1. The bacteria were grown in Luria-Bertani (LB) broth supplemented with the appropriate antibiotics [streptomycin (50 μg/mL) or tetracycline (12.5 µg/mL)] at 37 °C, with shaking. For infection assays, WT and Δ*escN* EPEC strains were transformed with a previously described pCX341 plasmid containing EspZ fused to the mature form of TEM-1 β-lactamase^55^. The Δ*escN* EPEC strain is a T3SS ATPase mutant that is devoid of T3SS activity.

**Table 1:** Species and plasmid used in this study.

	Description	Reference
**Species**		
Wild-type enteropathogenic *E. coli* (EPEC)	EPEC strain E2348/69, streptomycin resistant	^58^
EPEC Δ*escN*	Non-polar deletion of *escN*	^59^
*Vibrio cholerae*	*Vibrio cholerae* biotype EI-Tor serotype Inaba O1 In ET-122 (+)	^60^
*Citrobacter rodentium* DBS100	*C. rodentium* ATCC 51459	^61^
*Salmonella enterica*	Serovar Typhimurium (SL1344)	^62^
Enterohaemorrhagic *E. coli* (EHEC)	O157:H7 strain 86–24, nalidixic acid resistant	^42^
**Plasmid**		
pEspZ-TEM in pCX341	EspZ fused to β-lactamase	^55^

### Bacterial co-culture

WT EPEC, *V. cholerae*, and *E. coli* DH10B cultures were grown separately overnight at 37 °C (EPEC and DH10B) or at 30 °C (*V. cholerae*) in LB broth. The overnight cultures were then each diluted 1:40 into mixture of 1:1 (v/v) DMEM:plain LB medium and grown together in a tissue culture incubator (with 5% CO_2_) statically for 6 h, and their optical density at 600 nm (OD_600_) was measured. The cultures were centrifuged at 18000 × *g* for 10 min to collect the bacteria, the pellets were dissolved in SDS-PAGE sample buffer, and the supernatants were separated out and then filtered through a 0.22-μm low protein binding filter. The volumes of the supernatants and the bacterial pellets were normalized according to the OD_600_ of the bacterial cultures to ensure equal loading of the samples. To precipitate the proteins secreted into the culture medium, the supernatants were treated with 10% (v/v) trichloroacetic acid overnight at 4 °C.

### T3SS activity assay

EPEC strains were grown overnight at 37 °C in LB broth with appropriate antibiotics. The cultures were diluted 1:40 in either DMEM or in 1:1 (v/v) DMEM:plain LB supplemented with either 0.25–1% (v/v) DMSO or various concentrations of CAI-1 and grown in a tissue culture incubator (with 5% CO_2_) statically for 6 h; the optical density at 600 nm (OD_600_) of the cultures was measured. The cultures were centrifuged at 18000 × *g* for 10 min to collect the bacteria, the pellets were dissolved in SDS-PAGE sample buffer, and the supernatants were separated out and then filtered through a 0.22-μm low protein binding filter. The volumes of the supernatants and the bacterial pellets were normalized according to the OD_600_ of the bacterial cultures to ensure equal loading of the samples. To precipitate the proteins secreted into the culture medium, the supernatants were treated with 10% (v/v) trichloroacetic acid overnight at 4 °C. The secreted proteins were analyzed on 12% SDS-PAGE gels and stained with InstantBlue (Expedeon) or analyzed by western blotting.

To test the effect of bacterial supernatant fractions on EPEC T3SS activity, *V. cholerae* and *E. coli* DH10B were grown separately in LB broth overnight at 30 °C and 37 °C, respectively. The cultures were then centrifuged at 10000 × *g* for 5 min; supernatants were collected, centrifuged at 18000 × *g* for 10 min to remove the remaining cell debris and filtered through 0.22-μm filters. WT EPEC was inoculated 1:40 into 1:1 mixtures of either DMEM:plain LB or into DMEM:filtered bacterial supernatants (*V. cholerae* or *E. coli* DH10B) and continued as described above for evaluating T3SS activity in the presence of CAI-1.

The T3SS activity of EHEC was evaluated in a similar manner to that described for EPEC. We typically cultured 4-mL cultures for EPEC strains and 8-mL cultures for EHEC, which secreted much smaller amounts of proteins than EPEC.

T3SS activity of the *Salmonella* pathogenicity island 1 (SPI-1) was determined as described previously^43^. Briefly, the bacteria were grown in LB broth overnight at 37 °C. The cultures were diluted 1:40 into fresh LB, which approximates SPI-1-inducing conditions, supplemented with either 0.25% (v/v) DMSO or 25 μM CAI-1 and grown for 4 h. Then, the culture supernatants were collected and filtered, and their protein content was precipitated with 10% (v/v) trichloroacetic acid overnight at 4 °C. The samples were then centrifuged at 16000 × *g* for 30 min and washed with cold acetone, and the precipitated proteins were then dissolved in SDS-PAGE sample buffer.

### Western blotting

Samples were subjected to SDS-PAGE and transferred to nitrocellulose or PVDF membranes. Blots were blocked for 1 h in 5% (w/v) skim milk-PBST (0.1% Tween 20 in PBS) and then incubated with the primary antibody diluted in 5% skim milk-PBST for 1 h at room temperature. The optimal dilution for each antibody was calibrated individually. The secondary antibodies were diluted in 5% skim milk-PBST, incubated with the blots for 1 h at room temperature and detected with ECL reagents. The following commercial antibodies were used: mouse anti-DnaK (Abcam), mouse anti-JNK (BD Pharmingen), and mouse anti-actin (MPBio). Antibodies directed against T3SS components were a generous gift from Prof. B. Brett Finlay (University of British Columbia, Canada) and included mouse anti-EspA, mouse anti-EspB, rat anti-EscJ, mouse anti-Tir, and rabbit anti-SigD. Secondary antibodies were HRP-goat anti-mouse (Abcam) and HRP-goat anti-rat (Jackson ImmunoResearch).

### Chemical synthesis

Full synthetic methodologies to obtain racemic CAI-1, based on the synthesis of deuterated CAI-1^56^, are described in the Supplementary Methods.

### EPEC cultures

EPEC was grown overnight in LB in a shaking incubator at 37 °C. Bacterial growth was then measured under semi-optimal T3SS-inducing conditions by inoculating WT EPEC into pre-warmed 1:1 (v/v) DMEM:plain LB containing 0.25% (v/v) DMSO or 25 µM CAI-1. Bacterial growth was measured by following the OD_600_ of the cultures growing statically in a tissue culture incubator (with 5% CO_2_). At least three independent experiments were conducted, and average values are presented.

### RNA extraction and qPCR analysis

Bacteria were grown overnight in LB broth in a shaking incubator at 37 °C. WT EPEC was diluted 1:50 into pre-warmed 1:1 (v/v) DMEM:plain LB, while WT EHEC was diluted 1:50 into pre-warmed DMEM supplemented with either 0.25% (v/v) DMSO or 25 µM CAI-1 and grown statically in a tissue culture incubator (with 5% CO_2_) for 2 h (early exponential growth phase). *Salmonella* was diluted 1:50 into fresh LB medium supplemented with either 0.25% (v/v) DMSO or 25 µM CAI-1 and grown in a shaking incubator at 37 °C. Bacteria (5 × 10^8^) were collected and subjected to RNA extraction with the NucleoSpin Bacterial RNA isolation kit according to the manufacturer’s guidelines (Macherey-Nagel). From each sample, 200 ng of RNA were taken for cDNA synthesis by ProtoScript II First Strand cDNA Synthesis Kit (NEB) using random primer mix. cDNA was examined for genomic DNA contaminations, subjected to additional DNase I treatment, and extracted using TRIzol reagent when needed. The sequences of the primers used for the qPCR experiments are presented in Table 2. Melting curve analysis was used to ensure the specificity of each primer pair. RT-qPCR reactions with the cDNA of the examined samples and the primers to SYBR Green I mix (Roche) were analyzed in the LightCycler 480 instrument (Roche). The reaction conditions for amplification were: 1 cycle at 95 °C for 10 min, 40 cycles of 95 °C for 15 s, cooling to 60 °C for 10 s, followed by 72 °C for 10 s while monitoring fluorescence. The obtained data was analyzed by LightCycler 480 software to extract the critical threshold (*C*_*T*_) values. The expression levels of the target genes of the different treatments were normalized to the *rpoA* housekeeping gene and compared using the relative quantification method. Real-time data are presented as the fold change in expression levels.

**Table 2:** Primers used for real-time-PCR.

Gene	Primer name	Primer sequence	Reference
*rpoA*	qPCR_rpoA_F qPCR_rpoA_R	GGCGCTCATCTTCTTCCGAAT CGCGGTCGTGGTTATGTG	Modified based on ref.^63^
*espB EPEC*	qPCR_EspB_F qPCR_EspB_R	GGCTCTTTTGCTGCCATTAATAGC TCTGCTGCATCTGCAATACC	This study
*espA EPEC*	qPCR_EspA_F qPCR_EspA_R	GTGCGAATGCGAGTACTTCGAC TTGCAGCCTGAAAAACACCGAGT	This study
*tir EPEC*	qPCR_Tir_F qPCR_Tir_R	GGACCCTCTGCATTTCGTGTTG GTCCCCCGGTAAAAACAAATCTG	This study
*escJ EPEC*	qPCR_EscJ_F qPCR_EscJ_R	GCAAGCACTGTTGCTATCCA GCTGGGTGGGAAAATAACCT	This study
*espB EHEC*	qPCR_EspBH_F qPCR_EspBH_R	GCTTCGGAGAGTACGACCG CGGCCTGCTGAATCTGATAGC	This study
*espA EHEC*	qPCR_EspAH_F qPCR_EspAH_R	CCACGGCACAAAAGATGGC CCGCCTTCACTGTTTGCAG	This study
*tir EHEC*	qPCR_TirH_F qPCR_TirH_R	CGCCTGTAAGGAATTCTATGGC GCCTGTTAAGAGTATCGAGCGG	This study
*sipB*	qPCR_SipB_F qPCR_SipB_R	GGAACAAAGTCCGGCGAGAG TGAGACAGCGAAACATCGCC	^64^
*sipC*	qPCR_SipC_F qPCR_SipC_R	AAACCCAGTTACGCGAGCAG TGCCGTCGTTTTAGCTGCAT	^64^
*avrA*	qPCR_AvrA_F qPCR_AvrA_R	TGTTGAGCGTCTGGAAAGTG CAGATTCAACGCCTTCCATT	^65^

### Translocation assay

Translocation of TEM-1 fusions into HeLa cells was carried out as previously described^40,57^, with some modifications. Briefly, a day before the experiment, HeLa cells were seeded in black 96-well plates at a concentration of 2 × 10^4^ cells/well, and WT EPEC and EPEC Δ*escN* strain expressing the EspZ-TEM-1 fusion protein were grown overnight at 37 °C in LB. The bacteria were diluted 1:50 into DMEM containing 0.25% (v/v) DMSO or 25 µM CAI-1 for 2.5 h to OD_600_ of 0.1 under T3SS-inducing conditions before being used to infect HeLa cells at a multiplicity of infection of 1:100. Thirty minutes after the addition of the bacteria to the HeLa cells, EspZ-TEM-1 expression was induced by 1 mM of isopropyl β-d-1-thiogalactopyranoside (IPTG) for an additional hour. Cell monolayers were then washed twice with HBSS (Biological Industries) and incubated for 1 h at room temperature with freshly prepared CCF2/AM (Invitrogen). The plates were excited at 405 nm, and emissions at 460 nm and 530 nm were recorded (SpectraMax Paradigm; Molecular Devices). Relative TEM-1 translocation efficiency was expressed as the emission ratio of the cleaved CCF2 (blue; 460 nm) to original CCF2 (green; 530 nm) according to the manufacturer’s instructions. Three independent experiments were performed in triplicate and analyzed.

To confirm the translocation ability of EPEC, by using an additional translocation assay, we modified the protocol previously described by Baruch *et al*.^41^. Briefly, HeLa cells were infected for 60 min with EPEC strains (WT and Δ*escN*) that had been pre-induced for 2 h for semi-optimal T3SS activity (pre-heated 1:1 DMEM:plain LB mixture, statically, in a CO_2_ tissue culture incubator). HeLa cells were then washed with PBS, collected, and lysed with RIPA buffer. The samples were centrifuged at maximum speed for 5 min to remove unlysed cells, and the supernatants were collected, mixed with SDS-PAGE sample buffer, and subjected to western blot analysis with anti-JNK and anti-actin antibodies (loading control). Uninfected samples and the Δ*escN* mutant strain infected samples were used as negative controls.

## Supplementary information

Supplementary Information

## Data Availability

No datasets were generated or analyzed during the current study.
