# Supplementary material for: Vibrio cholerae autoinducer-1 enhances the virulence of enteropathogenic Escherichia coli
Source: Sci Rep. 2019 Mar 11;9:4122. doi: 10.1038/s41598-019-40859-1 (PMC6411865; doi:10.1038/s41598-019-40859-1)
Supplement: Supplementary file 1 — Supplementary Information [file 41598_2019_40859_MOESM1_ESM.docx]

***Vibrio cholerae* autoinducer-1 enhances the virulence of enteropathogenic *Escherichia coli***

### Orna Gorelik^1^, Niva Levy^2^, Lihi Shaulov^1^, Ksenia Yegodayev^1^, Michael M. Meijler^2^ & Neta Sal‑Man^1*^

^1^The Shraga Segal Department of Microbiology, Immunology and Genetics, Faculty of Health Sciences, Ben-Gurion University of the Negev, Beer-Sheva, Israel.

^2^The Department of Chemistry and the National Institute for Biotechnology in the Negev, Ben-Gurion University of the Negev, Beer-Sheva, Israel

*** Corresponding author:** salmanne@bgu.ac.il,

P.O.B. 653 Beer-Sheva 84105, Israel.

Phone (972) 86477295; Fax (972) 86277162

**Supplementary Information**

**CAI-1 synthesis** - Racemic CAI-1 was synthesized according to a previously reported^1^ procedure with minor modifications (Scheme S1).

2-nonyl-1,3-dithiane **-** 1,3-dithiane was suspended in freshly dried THF (0.5 M). A solution of *n*-butyllithium (1.6 M, 1.1 equivalents) was added at -78 °C, and the solution was stirred for 1.5 h at -5 °C. The reaction mixture was cooled to -78 °C and 1-bromononane in freshly dried THF (2.0 M) was added to the dithiane anion. The solution was slowly warmed to room temperature over 5 h. The reaction was then quenched with NH_4_Cl_(aq)_, extracted with EtOAc, dried over MgSO_4_, filtered and evaporated in vacuo. The product was purified using silica gel column chromatography, eluting with a gradient of toluene/hexane (1:19) to afford a pale yellow oil (67%). ^1^H NMR (400MHz, CDCl_3_) δ ppm 4.04 (t, *J =* 6.90 Hz, 1H), 2.85 (m, 4H), 2.11 (m, 1H), 1.86 (m, 1H), 1.50 (m, 2H), 1.26 (m, 14H), 0.88 (t, J=6.90, 3H), ^13^C NMR (400 MHz, CDCl_3_) δ ppm 47.7, 35.5, 31.9, 30.5, 29.5, 29.4, 29.3, 29.2, 26.6, 26.1, 22.7, 14.1. GCMS: calcd for C_13_H_26_S_2_, 246.15; found: 246.1.

1-(2-nonyl-1,3-dithian-2-yl)propan-1-ol **-** The alkylated dithiane was purified immediately prior to use by column chromatography. This dry dithiane was then suspended in freshly dried THF (0.2 M) and TMEDA (1 equivalent). A solution of *n*-butyllithium (1.6 M, 1.1 equivalents) was then added dropwise at -78 °C. The reaction mixture was stirred at 15 °C for 3 h, after which propionaldehyde (1 equivalent) in THF (0.2 M) was added dropwise. The reaction was stirred overnight, allowing it to reach room temperature. The reaction was then quenched with brine and saturated NH_4_Cl_(aq)_, extracted with diethyl ether, dried over MgSO_4_, filtered and evaporated in vacuo. The product was purified using silica gel column chromatography eluting with ethyl acetate/hexane (1:9) to afford a yellow oil (47%). ^1^H NMR (400 MHz, CDCl_3_) δ ppm 3.89 (dd, *J =* 1.67 Hz, 10.06 Hz, 1H), 2.99 (tdd, *J =* 3.17 Hz, 11.64 Hz, 14.65 Hz, 2H), 2.76 (s, 1H, -OH), 2.62 (m, 2H), 2.04 (m, 2H), 1.84 (m, 2H), 1.71 (m, 2H), 1.55 (m, 2H), 1.41 (m, 2H), 1.26 (m, 14H), 1.10 (t, J=7.37, 3H), 0.86 (t, J=7.12, 3H), ^13^C NMR (400 MHz, CDCl_3_) δ ppm 73.5, 59.3, 34.6, 31.9, 30.2, 29.6, 29.4, 29.3, 26.1, 25.1, 24.6, 23.3, 22.7, 14.1, 12.4 GCMS: calcd for C_16_H_32_S_2_O_2_, 304.19; found: 304.1.

3-hydroxytridecan-4-one **-** The dialkylated dithiane was suspended in a mixture of acetonitrile (0.4 M) and NaHCO_3(aq)_ (0.1 M). Iodine crystals (4 equivalents) were added portion wise at 0 °C. The reaction was stirred for 1 h at room temperature after which the reaction was diluted with diethyl ether and quenched with saturated Na₂S₂O₃_(aq)_ and saturated NaHCO_3(aq)_. The aqueous phase was extracted with diethyl ether. The organic phases were combined, dried over MgSO_4_, filtered and evaporated in vacuo, and purified using silica gel column chromatography, eluting with ethyl acetate/hexane (1:19) to afford the CAI-1 as a pale yellow solid (97%). ^1^H NMR (400 MHz, CDCl_3_) δ ppm 4.15 (td, *J =* 4.45 Hz, 6.77 Hz, 1H), 3.50 (d, *J =* 4.92 Hz 1H), 2.45 (m, 2H), 1.90 (m, 1H), 1.61 (m, 4H), 1.26 (m, 14H), 0.93 (t, J=7.40, 3H), 0.88 (t, J=6.88, 3H), ^13^C NMR (400 MHz, CDCl_3_) δ ppm 212.5, 37.9, 31.9, 29.4, 29.4, 29.3, 26.8, 23.6, 22.7, 14.1, 8.9. GCMS: calcd for C_16_H_26_O_2_, 214.19; found: 214.1.

**Scheme S1**: Synthesis of CAI-1: a.) *n*-BuLi, -78 ^o^C, THF then 1-bromononane b.) *n*-BuLi, -78 ^o^C, THF; then propionaldehyde, -78 ^o^C, THF c.) I_2_, 80% aqueous MeCN, 0 ^o^C

**Motility assay** – WT EPEC and *Citrobacter rodentium* were grown overnight at 37 °C in LB broth. Five microlitres from each culture were inoculated at the center of semi-solid agar plates containing 1% Tryptone, 0.5% NaCl, 0.2% agar and either 0.5 % (v/v) DMSO or 50 µM CAI-I. Bacterial motility was measured by determining the diameter of the circle formed by the bacteria after 8 h at 37 °C. Average values of four independent experiments are presented in Figure S3. *C. rodentium* is non-motile bacterium and was therefore used as a negative control.

**Bioluminescence (LuxR) assay** – The presence of CAI-1 in the medium was examined by following the light production by *V. cholerae* harbouring the luxCDABE operon (reporter strain). WT *V. cholerae*, *V. cholerae* harbouring the luxCDABE operon (reporter strain) and *E. coli* DH10B were grown overnight at 30 °C (*V. cholerae* strains) or 37 °C (*E. coli* strain) in LB broth. Fifty microlitres of the reporter strain (diluted 1:5 into fresh LB) was added to 150 μL of either WT *V. cholerae* or *E. coli* DH10B supernatants. Diluted cultures were grown at 30 °C with aeration, and light production and OD_600_ were measured every 30 min (TECAN Infinity 200pro). Plain LB and 10 μM CAI-1 were used as negative and positive controls, respectively. Relative units were defined as luminescence divided by OD_600_. The results are presented as average values of three replicates.

**Biofilm formation assay** – The biofilm assay was performed as described previously^2^. Briefly, overnight cultures were grown at 37 °C in LB. The cultures were then adjusted to 1.0 OD_600_ and diluted 1:10 in fresh LB in a 96-well polystyrene plate. The plate was incubated statically at 26 °C for 48 h. Following the incubation period, the plate was washed three times with phosphate buffered saline (PBS), dried, and stained with 0.1 % crystal violet for 20 min. The OD_570_ values were determined in a microplate reader after solubilization of the dye with 95% ethanol. A non-biofilm forming strain (*E. coli* HB101) was used as a negative control.


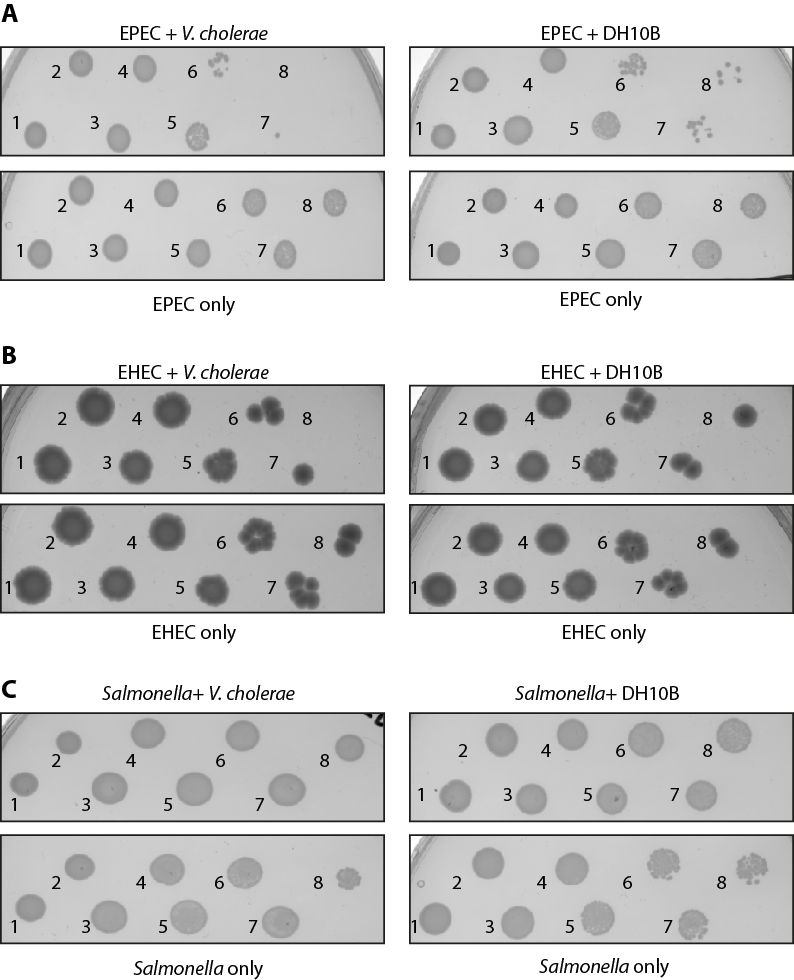


**Figure S1**: (A) To monitor the survival of EPEC when grown in pure culture or in co-culture with *V. cholerae* or *E. coli* DH10B, we grew the bacteria in Transwells (Merck, MultiScreen filter plates 0.22 μm) for 6 h in 1:1 (v/v) DMEM:plain LB. EPEC was then collected and spotted at 10-fold serial dilutions (where 1 indicates the original sample) on an LB agar plate supplemented with streptomycin. The plates were incubated overnight at 37 °C. The growth of EHEC (B) and *Salmonella* (C) in the presence and absence of *V. cholerae* or *E. coli* DH10B was monitored as described in panel A. EHEC was plated on an LB agar plate supplemented with nalidixic acid, while *Salmonella* was plated on an LB agar plate supplemented with streptomycin.


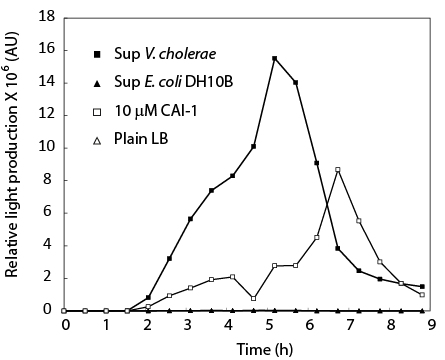


**Figure S2**: Relative light production as means to determine the level of AIs found in the supernatants of WT *V. cholerae* or *E. coli* DH10B. While no light production was observed in the presence of *E. coli* DH10B supernatant, a high signal was detected in the presence of WT *V. cholerae* supernatant. In the presence of 10 μM synthetic CAI-1, the reporter strain showed light production, while no signal was observed when it was grown in LB broth alone. Due to the low levels of light production observed for plain LB and the supernatant of *E. coli* DH10B, the symbols for plain LB are hidden by the symbols of *E. coli* DH10B supernatant. The Figure presents average values of three replicates.


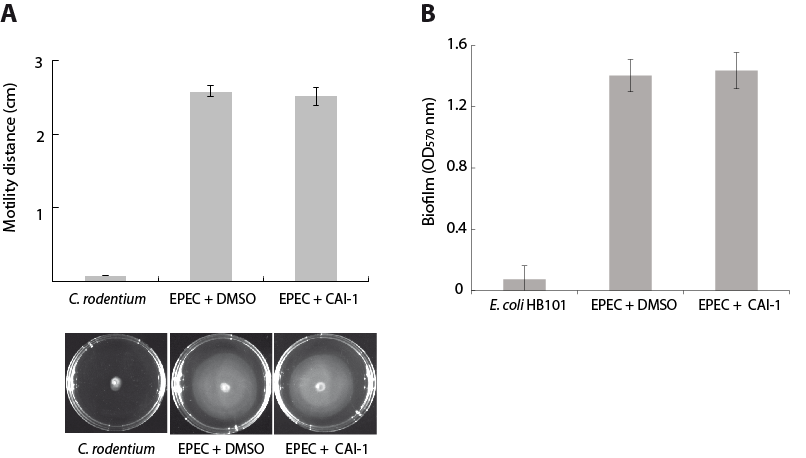


**Figure S3**: **CAI-1 does not affect EPEC motility or biofilm formation.** (A) WT EPEC motility was determined by spotting 5-μL overnight bacterial cultures on semi-solid agar plates, containing either 0.5 % (v/v) DMSO or 50 μM CAI-1, and allowing the bacteria to spread for 8 h at 37 °C. The diameter of the circle formed by the bacteria was measured in four independent experiments, and average diameter is presented together with pictures of representative plates. We observed that the bacteria formed similar sized circles around the original spot in the presence of 50 μM CAI-1 or DMSO supplement alone, thus suggesting that CAI-1 had no effect on the motility of WT EPEC, even in the presence of twice the concentration used for the functional assays. (B) The bacterial strains were incubated in LB broth for 48 h at 26 °C and were considered to be biofilm producers when OD_570_ exceeded 0.5*.* EPEC was grown in the presence and absence of CAI-1 (25 μM) with the aim to evaluate the effect of CAI-1 on biofilm formation.  *E. coli* HB101 was used as a negative control for the biofilm producer strain. Data are taken from a representative experiment. The bars represent standard deviation.

1 Kelly, R. C. *et al.* The *Vibrio cholerae* quorum-sensing autoinducer CAI-1: analysis of the biosynthetic enzyme CqsA. *Nat. Chem. Biol.* **5**, 891-895, doi:10.1038/nchembio.237 (2009).

2 Nascimento, H. H., Silva, L. E., Souza, R. T., Silva, N. P. & Scaletsky, I. C. Phenotypic and genotypic characteristics associated with biofilm formation in clinical isolates of atypical enteropathogenic *Escherichia coli* (aEPEC) strains. *BMC Microbiol.* **14**, 184, doi:10.1186/1471-2180-14-184 (2014).

Full-length blots

**Figure 1**:

The gel of the supernatant was cut after transfer and blot separately using anti-EspA (lower) and anti-EspB (upper) antibodies.

The gel of the bacterial pellet was cut after transfer and blotted separately using anti-Tir (upper) and anti-EscJ (lower) antibodies.

Anti-DnaK (lower) antibody:

**Figure 2:**

The gel of the supernatant was cut after transfer and blot separately using anti-EspA (lower) and anti-EspB (upper) antibodies.

The gel of the bacterial pellet was cut after transfer and blotted separately using anti-Tir (upper) and anti-EscJ (lower) antibodies.

Anti-DnaK (lower) antibody:

**Figure 3:**

The gel of the supernatant was cut after transfer and blot separately using anti-EspA (lower) and anti-EspB (upper) antibodies.

The gel of the bacterial pellet was cut after transfer and blotted separately using anti-Tir (upper) and anti-EscJ (lower) antibodies.

Anti-DnaK (lower) antibody

**Figure 6:**

Full-length gel of anti-actin

**Figure 7**

anti-EspA for EHEC:


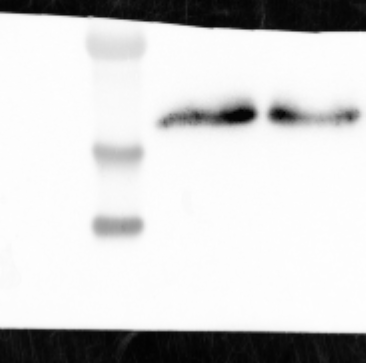


anti-SigD for *Salmonella* samples:
